# Electrochemical Genosensing of Overexpressed GAPDH Transcripts in Breast Cancer Exosomes

**DOI:** 10.1021/acs.analchem.2c04773

**Published:** 2023-01-23

**Authors:** Arnau Pallares-Rusiñol, Silio Lima Moura, Mercè Martí, Maria Isabel Pividori

**Affiliations:** † Grup de Sensors i Biosensors, Departament de Química, Universitat Autònoma de Barcelona, Bellaterra 08193, Spain; ‡ Biosensing and Bioanalysis Group, Institute of Biotechnology and Biomedicine, Universitat Autònoma de Barcelona, Bellaterra 08193, Spain

## Abstract

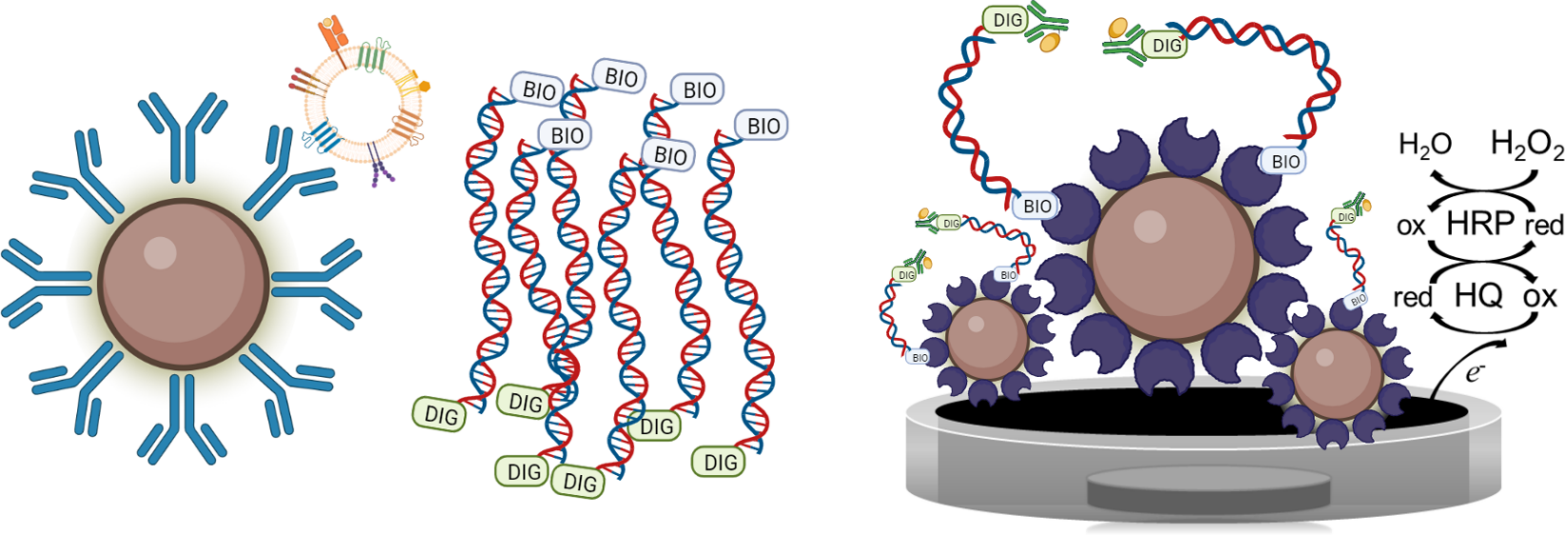

Exosomes are receiving highlighted attention as new biomarkers for the detection of cancer since they are profusely released by tumor cells in different biological fluids. In this paper, the exosomes are preconcentrated from the serum by immunomagnetic separation (IMS) based on a CD326 receptor as a specific epithelial cancer-related biomarker and detected by glyceraldehyde-3-phosphate dehydrogenase (GAPDH) transcripts. Following the lysis of the captured exosomes, the released GAPDH transcripts are amplified by reverse transcription polymerase chain reaction (RT-PCR) with a double-tagging set of primers on poly(dT)-modified-MPs to increase the sensitivity. The double-tagged amplicon is then quantified by electrochemical genosensing. The IMS/double-tagging RT-PCR/electrochemical genosensing approach is first demonstrated for the sensitive detection of exosomes derived from MCF7 breast cancer cells and compared with CTCs in terms of the analytical performance, showing an LOD of 4 × 10^2^ exosomes μL^–1^. The genosensor was applied to human samples by immunocapturing the exosomes directly from serum from breast cancer patients and showed a higher electrochemical signal (3.3-fold, *p* < 0.05), when compared with healthy controls, suggesting an overexpression of GAPDH on serum-derived exosomes from breast cancer patients. The detection of GAPDH transcripts is performed from only 1.0 mL of human serum using specific magnetic particles, improving the analytical simplification and avoiding ultracentrifugation steps, demonstrating to be a promising strategy for minimal invasive liquid biopsy.

## Introduction

Breast cancer is a highly lethal malignancy and the most commonly diagnosed cancer among women, with an estimated over 2 million new cases in 2020.^1^ Most of the currently available technologies for breast cancer diagnosis are based on imaging techniques.^2^ In high-income countries, breast cancer is detected at stages I and II in 70% of women, while 20–50% in low-income countries. Moreover, the time delay that exists between diagnosis and treatment is about 4 to 6 weeks in high-income countries, but it can be as long as 8 months in low- and middle-income countries.^3^ The use of biomarkers related with breast cancer in liquid biopsies for early identification of individuals could potentially bridge this gap in low-resource settings, by reducing the technical requirements and operational costs. The routine clinical diagnosis in liquid biopsies is based on enzyme-linked immunosorbent assay (ELISA) methodologies,^4^ existing many commercial kits targeting breast cancer-related biomarkers (e.g., BRCA1 ELISA kit, from MBS; CA15-3 ELISA kit, from Abcam; or BCAR ELISA kit, from Biogen). Besides, other techniques aim for the detection of circulating tumor cells (CTCs),^5^ considered one of the most significant breast cancer-related biomarkers. CellSearch^6,7^ is the first and the only CTC-based assay commercially available and approved by the US Food and Drug Administration (FDA). CellSearch enriches CTCs using magnetic particles containing antibodies against the Epithelial Cell Adhesion Molecule (EpCAM) (also known as CD326). EpCAM is a cell-surface glycoprotein that is known to be highly expressed in epithelial carcinomas, including breast cancer and prostate cancer.^8^ However, the clinical use of CTCs is limited by their scarcity in the peripheral blood (1 CTC/10^5–6^ blood cells).^9^

Exosomes^10^ (30–200 nm in diameter) are receiving highlighted attention as new biomarkers for the detection of cancer in early stages.^11,12^ Exosomes are intercellular shuttle-like vesicles with molecular cargo as mRNA, microRNA, DNA, lipids, and proteins.^13^ Most cell types, including normal and tumor cells, release exosomes in many different biological fluids such as blood, plasma, serum, or urine, among others.^13^ It is known that a single cell can release many exosomes per hour into the extracellular space,^14^ at an increased rate by tumor cells. The high number of exosomes that can be released by a single tumor cell reveals the strong potential application of exosomes as an alternative biomarker for early diagnostics, overcoming the most challenging limitation that presents CTC assays: their very low concentration in blood. Exosomes can potentially be used to detect the presence of tumor cells and deposits in the early stage of growth with a simple and minimally invasive procedure such as liquid biopsy.

In this work, it is described an electrochemical genosensor for the detection of glyceraldehyde-3-phosphate dehydrogenase (GAPDH) gene transcripts, which we found to be overexpressed in breast cancer cells and exosome-derived human serum in breast cancer patients. The approach is based on immunomagnetic separation (IMS) of the exosomes using CD326 cancer-related biomarker, followed by amplification by double-tagging reverse transcription PCR of the GAPDH transcripts on poly(dT)-MPs. The integration of PCR with electrochemical genosensing was previously reported,^15^ as well as the further detection of the labeled DNA amplicons from double-tagging PCR^16,17^ and quadruple-tagging multiplex PCR.^18,19^ This approach is first optimized with breast cancer cell line MCF7 cells and exosomes obtained from a cell culture supernatant. Only 1.0 mL of human serum is used to specifically capture the exosomes on MPs which can be easily integrated on the biosensing device, improving the analytical simplification by avoiding ultracentrifugation. The electrochemical genosensing approach allows the quantitative measurement of transcripts with high sensitivity, robustness, and simplicity. To the best of the authors’ knowledge, this is the first study on the expression of GAPDH genes in exosomes from breast cancer patients.

## Experimental Section

### Instrumentation

Nanoparticle tracking analysis (NTA) was performed using the NanoSight LM10-HS system (NanoSight Ltd., Malvern, GB). The cryogenic transmission electron microscopy (TEM) images were collected by a Jeol JEM 2011 (JEOL USA Inc., MA, US) microscope. Flow cytometry was performed using BD FACSCANTO II (BD Biosciences, NJ, US) equipment. Mean fluorescence intensity (MFI) and beads count data were obtained by FlowJo analysis software (FlowJo LLC, BD Biosciences) of every sample-reading file. The confocal images were collected on the microscope Leica, TCS SP5 (Leica Microsystems, DE). SimpliAmp Thermal Cycler (Applied Biosystems, US) was used for the double-tagging reverse transcription polymerase chain reaction (RT-PCR) amplification. All electrochemical experiments were performed using an Autolab PGSTAT10 (Metrohm AG, CH) potentiostat/galvanostat electrochemical analyzer. A magneto-actuated graphite-epoxy composite (m-GEC) electrode as the working electrode (geometric area = 0.5 cm^2^), Ag/AgCl/KCl(sat.) as the reference electrode, a disc platinum counter electrode (geometric area = 3.0 cm^2^), and a standard 20-mL one compartment three-electrode cell was used in all experiments. The detailed preparation of the m-GEC electrodes has been extensively described by Pividori and co-workers.^16−19^

### Chemicals and Biochemicals

Tosyl-activated magnetic particles (MPs) (Dynabeads M450 Tosylactivated, ref. 14013), MPs modified with EpCAM antibody (antiCD326-MPs, Dynabeads Epithelial Enrich, ref. 16102), MPs modified with poly(dT) (polydT-MPs, Dynabeads Oligo(dT)25, ref. 61002), MPs modified with streptavidin (strep-MPs, Dynabeads MyOne Streptavidin T1, ref. 65601), mouse monoclonal antibody antiCD81 (ref. 10630D), and BCA protein assay kit (ref. 23225) were purchased from Thermo Fisher Scientific (MA, US). Mouse monoclonal antibody antiCD326 or EpCAM (ref. ab7504) and a goat anti-mouse IgG H&L (Cy5) (antimouse-Cy5, ref. ab97037) were purchased from Abcam (Cambridge, GB). Antidigoxigenin-horseradish peroxidase Fab fragments (antiDIG-HRP, ref. 11207733910) were purchased from Roche Diagnostics (Basel, CH).

The primers for the double-tagging PCR were selected for the specific amplification of GAPDH and were purchased from Sigma-Aldrich (Merck KGaA, DE). The sequence for the digoxigenin-modified forward primer (DIG-Fw) was 5′-[DIG] CTTCTTTTGCGTCGCCAG, while the sequence for the biotin-modified reverse primer (BIO-Rev) was 5′-[BIO] AGCCCCAGCCTTCTCCA. The primers were also checked in terms of secondary structures, to avoid hairpins, self, or cross dimers. All solutions, described in S1 (Supplementary Data), were prepared with Ultrapure water (Millipore System, resistivity 18.2 MΩ cm) and solutions used in RNA preparation were RNase-free by treatment with 0.1% DEPC.

### Cell Culturing, Exosome Isolation, and Purification from the MCF7 Cell Line

The exosomes were obtained from the MCF7 cell line (ATCC, ref. HTB-22), and the culture conditions are detailed in S2 (Supplementary Data). The MCF7 cells were used as a model of breast cancer. The exosomes were purified from the culture supernatant by differential ultracentrifugation according to Théry et al.^20^ with minor changes. Exosomes were resuspended in Tris 1× buffer (pH 7.4, 0.22 μm sterile-filtered) and stored at −80 °C. All the experimental data are provided in S2 (Supplementary Data).

### Characterization of the Exosomes Derived from MCF7 Breast Cancer Cell Lines

The size distribution and concentration of exosomes were measured by nanoparticle tracking analysis (NTA). The morphology was analyzed by cryogenic transmission electron microscopy (Cryo-TEM). The total protein concentration of exosomes samples was estimated by the BCA protein assay kit (Pierce BCA protein assay kit, ref. 23227, Thermo Fisher Scientific).

To set up the technical approach, the expression study of CD81, a tetraspanin general marker for exosomes, and CD326, a cancer-related epithelial receptor, on the MCF7 cell line and their derived exosomes was carried out by flow cytometry. In the case of the cells, the indirect labeling was performed by the incubation of specific antibodies antiCD81 and antiCD326, followed by labeling with the antimouse-Cy5 antibody (a far-red-fluorescent dye, excitation 647 nm, emission 665 nm). The labeled cells were resuspended in Tris 1× buffer solution containing 0.5% BSA solution.

In order to compare the expression on the exosomes, the same procedure of labeling was performed, but in this case, the exosomes were first immobilized on the surface of MPs due to their size and the resolution of the technique. To achieve that, exosomes were covalently immobilized on tosyl-activated MPs, as detailed in S3 and Figure S1, panel A (Supplementary Data). Then, indirect labeling was performed first incubating with antiCD81 or antiCD326, followed by incubation with the antimouse-Cy5 antibody. In parallel, the same batch of cells and exosomes analyzed by flow cytometry was subjected to confocal microscopy imaging for the study of the binding pattern of antibodies. In the case of cells, nuclear DNA was stained with Hoechst dye (a blue-fluorescent dye, emission wavelength 490 nm) before labeling with antibodies. Further experimental details and incubations are described in S4 (Supplementary Data).

### Immunomagnetic Separation, Double-Tagging Reverse Transcription PCR of GAPDH Transcripts, and Electrochemical Genosensing

The procedure was evaluated on cells and exosomes derived from the MCF7 breast cancer cell line as a model. Briefly, it consists of (i) immunomagnetic separation of the cells/exosomes, (ii) double-tagging reverse transcription PCR of GAPDH transcripts, and (iii) electrochemical genosensing. After the optimization, the approach was used for the evaluation of exosomes in human serum from breast cancer individuals.

**Figure 1:**
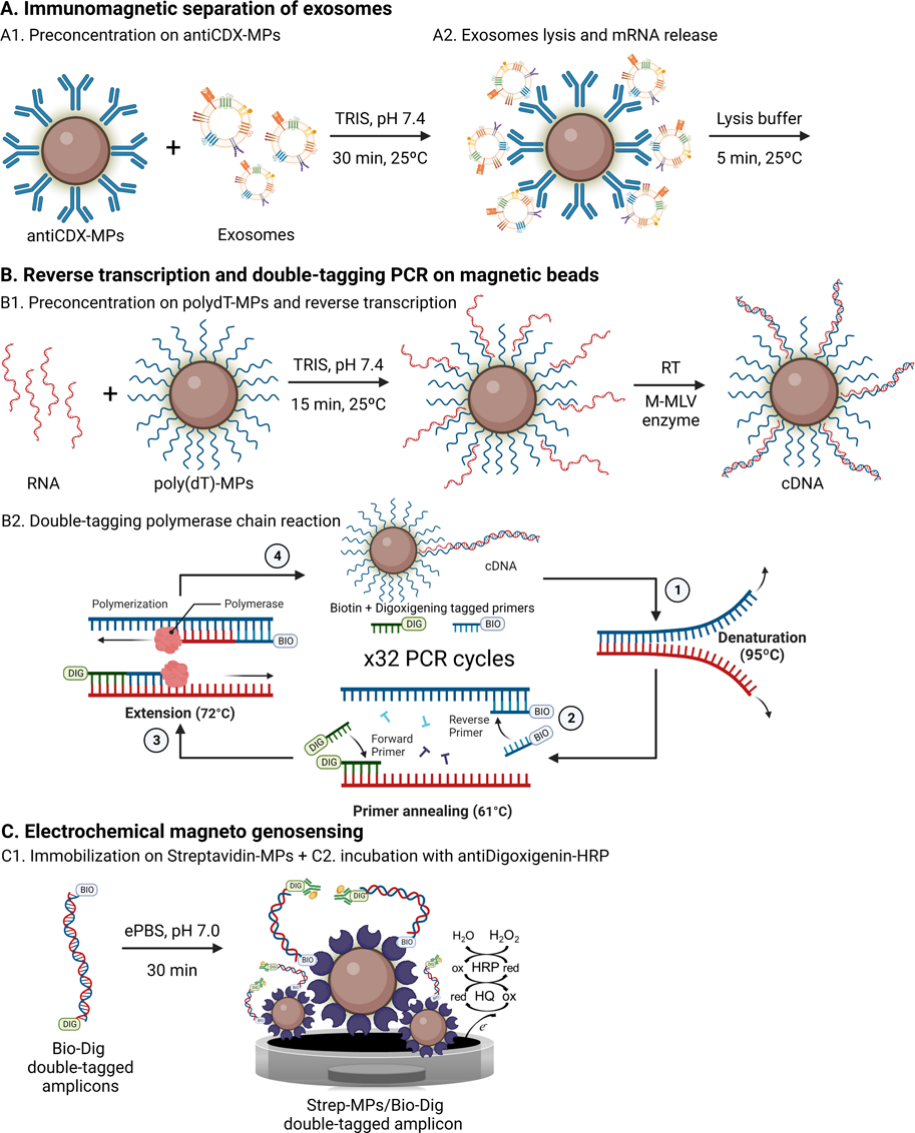
Schematic representation for the detection of GAPDH expression by the immunomagnetic separation of exosomes (panel A1) and lysis (panel A2); mRNA extraction with poly(dT)-MPs and reverse transcription (panel B1), and double-tagging PCR (panel B2); and electrochemical magneto genosensing with amperometric readout (panel C). Created with BioRender.com.

This approach sequentially combines three different types of magnetic separations, as depicted in Figure 1. First, the method involves the cells or exosome preconcentration based on the specific separation with magnetic particles modified with the antiCDX antibody (CDX being either CD81 or CD326; Figure 1, panel A1). Then, they were lysed and the released messenger RNAs (Figure 1, panel A2) were captured by polydT-MPs based on the poly(A) tail followed by reverse transcription to obtain cDNA (Figure 1, panel B1). After that, the cDNA was amplified by double-tagging PCR on the magnetic beads (Figure 1, panel B2), using a double-tagging set of primers specific for GAPDH. During PCR, the cDNA is not only amplified but also labeled at the same time with biotin/digoxigenin (BIO/DIG) tags. Finally, the electrochemical magneto-genosensing was performed on streptavidin-magnetic particles as a support, based on the BIO tag through biotin–streptavidin interactions. The DIG tag was used for labeling with the antiDIG-HRP conjugate. The electrochemical readout of the double-tagged amplicons was based on peroxidase (HRP) enzymes as electrochemical reporters and performed on m-GEC electrodes, as previously reported^18^ (Figure 1, panel C). The experimental details are described in the next sections and further detailed in S5 (Supplementary Data).

#### Immunomagnetic Separation of the Cells and Exosomes

Cells (100 μL) (at different concentrations ranging from 50 to 5000 cells mL^–1^) or exosomes (from 100 to 4.0 × 10^4^ exosomes μL^–1^) were incubated with 1 × 10^6^ antiCDX-MPs (CDX being either CD81 or CD326) for 30 min at 25 °C while shaking, followed by washing with Tris 1× buffer containing 0.5% BSA. The content of the preconcentrated cells or exosomes on antiCDX-MPs was released by resuspending them on 1 mL of lysis/binding buffer and disrupted using a syringe.

#### Double-Tagging RT-PCR on Magnetic Beads

The mRNA extraction and purification on polydT-MPs based on the polyA tail of the transcripts was performed, followed by reverse transcription on polydT-MPs (Figure 1, panel B1). The lysate was incubated with 15 μL of polydT-MPs (75 μg, equivalent to 7.5 × 10^7^ MPs) for 15 min under gentle shaking at 25 °C, washed three times, and stored in ice. In order to obtain the cDNA, the retrotranscription (RT) was carried out on poly(dT)-MPs with Moloney Murine Leukemia Virus (M-MLV) reverse transcriptase. The RNA-poly(dT)-MPs were incubated with 10 nmol of dNTPs mix for 5 min at 65 °C and cooled on ice for 1 min. After that, a mix containing 200 nmol of DTT, 40 U of RNaseOUT inhibitor, and 1× First Strand Buffer was added and incubated at 37 °C for 2 min. Finally, 200 U of M-MLV reverse transcriptase were added and incubated for 50 min at 37 °C and 15 min at 70 °C for inactivating the reaction. The cDNA was stored at −21 °C until use.

The double-tagging polymerase chain reaction (PCR) was performed in 15 μL of the reaction mixture containing the cDNA (Figure 1, panel B2). Each reaction mixture contained 7.5 pmol of each primer (DIG-Fw and BIO-Rev), 3.75 nmol of each dNTPs, and 3 U of Taq polymerase. The reaction was carried out in a buffer with 7.5 mmol L^–1^ Tris buffer (pH 9.0), 5.0 mmol L^–1^ KCl, 2.0 mmol L^–1^ (NH_4_)_2_SO_4_, and 0.2 mmol L^–1^ MgCl_2_ as a cofactor of the enzyme. The reaction mixture was exposed to an initial step at 95 °C for 3 min followed by 32 cycles of 95 °C for 30 s, 61 °C for 30 s, 72 °C for 30 s, and a last step of 7 min at 72 °C. Negative controls for both the RT and PCR were performed as above, except adding mRNA or cDNA, respectively.

The performance of the double-tagging RT-PCR amplification was checked with 2% agarose gel electrophoresis in TAE buffer containing 1× GelRed dye. The DNA bands were visualized by UV transillumination. A single DNA band was obtained in all samples sized around 371 bp. To confirm that GAPDH was amplified, all bands were cut from the gel and purified with the GeneJET kit, and DNA sequencing was performed.

### RNA Integrity Analysis and DNA Sequencing

For the integrity analysis, the RNA from breast cancer cells and exosomes was extracted using the total exosome RNA and the protein isolation kit (Thermo Fisher Scientific, ref. 4478545) and analyzed with the Agilent RNA 6000 Nano Kit (ref. 5067-1511, Agilent) by Genomics Bioinformatics Service (Institute of Biotechnology and Biomedicine, UAB, ES). The DNA sequences of the PCR amplicons were obtained with an ABI Prism 3130XL Genetic Analyzer by the same GBS and were analyzed using Chromas v 2.6.6 (Technelysium Pty Ltd., Brisbane, QLD, AU) and Clustal Omega^21^ software to check the chromatograms and the alignment of both sequences.

### Electrochemical Magneto-Genosensing

Briefly, after the double-tagging RT-PCR, the BIO-tag was used for the immobilization of the amplicons on streptavidin-magnetic particles through the high affinity biotin–streptavidin interaction, while the DIG tag allowed the labeling by the antiDIG-HRP, in one 15 min step. The procedure comprised, as described in Figure 1, panel C: (a) the immobilization and preconcentration of the tagged amplicons on 7 × 10^7^ strep-MPs and (b) the incubation with the electrochemical reporters in one step for 15 min at RT, with 10 μL (130 mU) of antiDIG-HRP. Two washing steps with 500 μL of Tris 1× buffer for 2 min at RT were performed. After the incubation or washing step, a magnetic separator was positioned under the tubes until pellet formation on the tube side wall, followed by supernatant separation; (c) magnetic actuation on the m-GEC; and (d) amperometric readout using applying a potential of −100 mV (vs Ag/AgCl_sat_), under enzyme saturation conditions in ePBS buffer, upon the addition of hydroquinone and hydrogen peroxide. All experimental steps are described in detail in S7 (Supplementary Data).

The steady-state cathodic amperometric current (*I*_cat_, in μA) was used for the electrochemical signal plotted in all the figures. Different parameters of the electrochemical genosensing, such as the washing step time, the incubation time with the electrochemical reporter, the concentration of strep-MPs, and the electrochemical reporter, and finally, the procedure in one or two steps for electrochemical genosensing were previously optimized by our group.^18^

### Electrochemical Magneto-Genosensing of Transcripts from Exosomes of Breast Cancer Patients

Blood samples from healthy donors (*n* = 10, 5 men and 5 women, mean age 30/SD = 5) and breast cancer donors (*n* = 10, stage IV, all women, mean age 50/SD = 6) were obtained already anonymized from the Hospital del Mar (Barcelona, ES) and pooled for further separation of the exosomes. The work was carried out in accordance with the principles of voluntariness and confidentiality. The samples were treated as described in S7 (Supplementary Data). In this instance, the IMS of the exosomes from 1 mL of pooled and anonymized undiluted human serum (healthy and breast cancer patients) was directly performed on magnetic particles modified with the epithelial biomarker CD326 (antiCD326-MPs). The IMS involved the following steps: (i) IMS of the exosomes with antiCD326-MPs (containing 2 × 10^6^ MPs per tube) and 1.0 mL of human serum were simultaneously incubated for 30 min with gentle shaking at 25 °C, followed by washing with Tris 1× buffer containing 0.5% BSA. Then, the exosome-coated antiC326-MPs were resuspended with 100 μL of Tris 1× buffer, stored on ice, and immediately used for RNA extraction. All further steps were performed as described in the Experimental Section, including double-tagging reverse transcription PCR of GAPDH transcripts and electrochemical genosensing. The complete assay protocols, as well as the preparation of human serum from blood, are provided in S7 (Supplementary Data).

### Statistical Analysis

The statistical analyses and calculations were performed using GraphPad Prism 8 (San Diego, CA, US) while plots were represented using Origin Pro 2017 (Northampton, MA, US).

### Safety Considerations

All studies were performed in a biosafety cabinet, and all material decontaminated by autoclaving or disinfected before discarding in accordance with U.S. Department of Health and Human Services guidelines for level 2 laboratory Biosafety.^22^

## Results and Discussion

### Characterization of the Exosomes Derived from MCF7 Breast Cancer Cell Line

**Figure 2:**
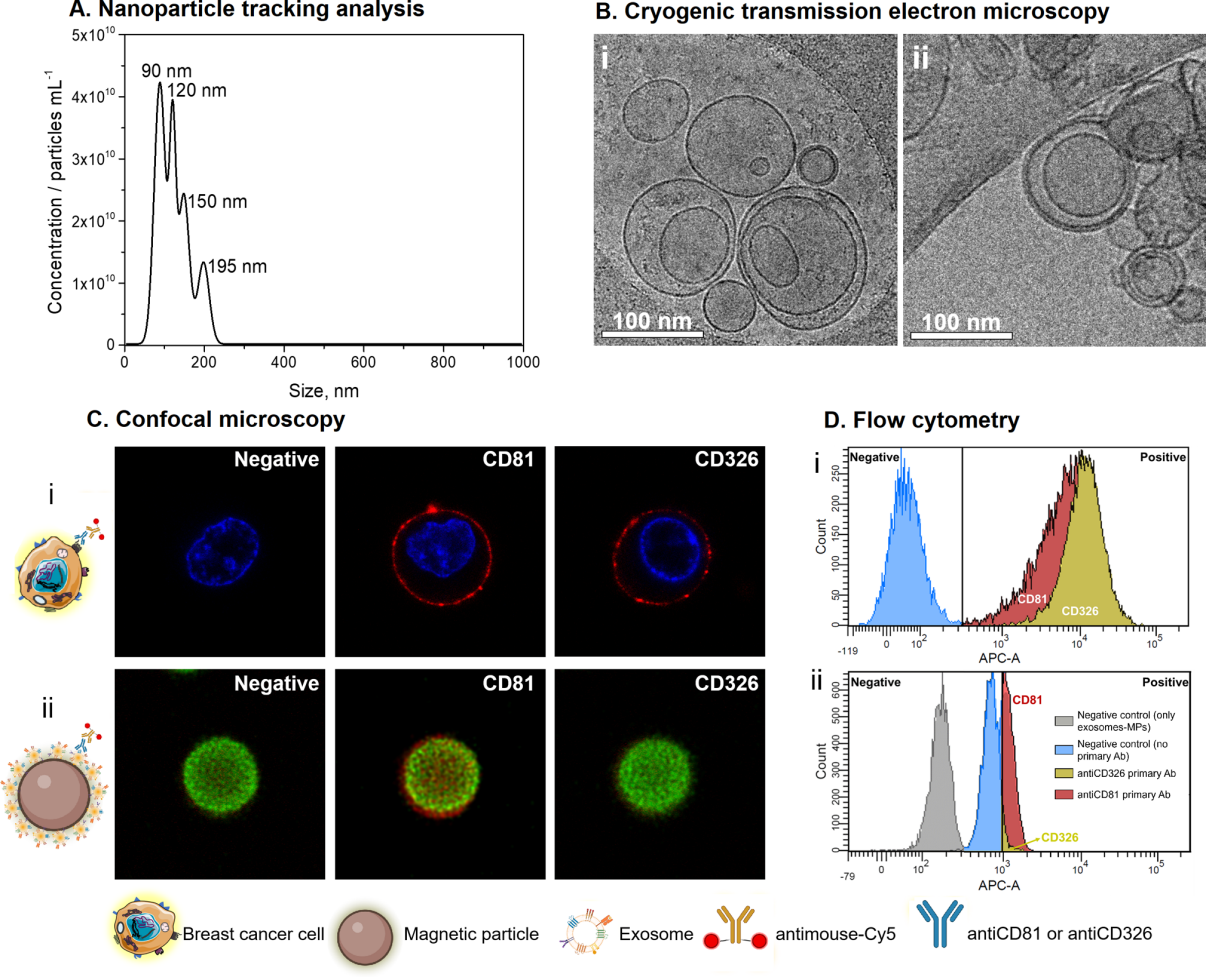
(A) Characterization by NTA of purified exosomes derived from the MCF7 breast cancer cell line. (B) Cryo-TEM images (i) and (ii) of purified exosomes at an acceleration voltage of 200 kV. (C) Confocal microscopy images and (D) flow cytometry study for the (i) MCF7 breast cancer cell line and (ii) their exosomes covalently immobilized on MPs. For confocal microscopy, DNA appears blue, magnetic particles in green color, while the exosome protein membrane in red color. For flow cytometry, in all cases, the negative controls (obtained with incubation with the secondary antimouse-Cy5) are shown in blue, while the positive CD326 and CD81 samples obtained with the incubation with the antiCD326 and antiCD81 primary antibodies, are shown in yellow and red, respectively. The negative control obtained with the exosomes-modified MPs without any labeling is shown in gray in panel D (ii).

An estimation of the size diameter distribution and the concentration of purified exosomes derived from MCF7 breast cancer cell lines was performed by nanoparticle tracking analysis (NTA). Figure 2, panel A, shows that the size diameter distribution of exosomes ranges from 30 up to 210 nm, which is represented by exosomes with 90, 120, 150, and 195 nm in diameter, in accordance with the expected size range for exosomes.^23^ Further information of size diameter distribution was obtained by Cryo-TEM. Micrographs of the exosome sample show well-shape exosomal vesicles with closed circular lipid bilayers (Figure 2, panel B) around 110 nm in diameter. Cryo-TEM micrographs also reveal the presence of some exosome aggregates.

Confocal microscopy demonstrated qualitatively that the CD81 and CD326 membrane receptors are well-expressed in the MCF7 breast cancer cell line (Figure 2, panel C(i)), as well as their expression on MCF7-derived exosomes covalently immobilized on magnetic particles (Figure 2, panel C(ii)). The intense green color of the magnetic particles was due to autofluorescence around 580 nm.^24^ Negligible nonspecific adsorption was observed (Figure 2, panel C(ii), negative). The CD81 tetraspanin was also shown with strong labeling in exosomes, although a poor labeling pattern was achieved for the CD326 biomarker.

Quantitative patterns of the MCF7 cell line were also studied by flow cytometry analyzing the expression to CD81 and CD326, as shown in Figure 2, panel D. The negative control in which the signal appears onto the left side confirms that there is a negligible (<0.1%) nonspecific reaction with the secondary antibody (antimouse-Cy5 antibody) with the MCF7 cells (Figure 2, panel D(i)). As expected, the percentage marker expression to CD81 and CD326 biomarkers was high as >95% for MCF7 cells (Figure 2, panel D(i)). The same CD81 and CD326 biomarkers on exosomes derived from the MCF7 breast cancer cell line were also studied by flow cytometry. As expected, exosomes covalently immobilized on magnetic particles (exosomes-MPs) highly expressed CD81, but CD326 showed a low expression pattern with this model (<5%) (Figure 2, panel D(ii)), in accordance with the results obtained by confocal microscopy. Tetraspanins are the most frequently identified proteins in exosomes and are considered classical markers. Comparing the expression levels of cells and their derived exosomes, and according to many studies,^25−27^ cell-membrane biomarkers are not always identically expressed in the cells as well as in their derived exosomes. These data suggest that the exosomal molecular profile needs to be carefully assessed to achieve a better experimental approach design.

Regarding the genetic material in MCF7 cells and exosomes, it was characterized by RNA integrity analysis, obtaining significantly different patterns, as shown in S6 (Supplementary Data).

### Immunomagnetic Separation, Double-Tagging Reverse Transcription PCR of GAPDH Transcripts, and Electrochemical Genosensing

First, all steps from the proposed IMS/double-tagging RT-PCR/electrochemical genosensing detection method were tested and optimized with MCF7 cells and purified exosomes, as described in S5 (Supplementary Data). The DNA sequences of the PCR amplicons were also obtained. The genome sequence for the *Homo sapiens* mRNA GAPDH transcript was identified by using BLAST software.^28^ Further details are provided in S6 and Figure S3 (Supplementary Data).

**Figure 3:**
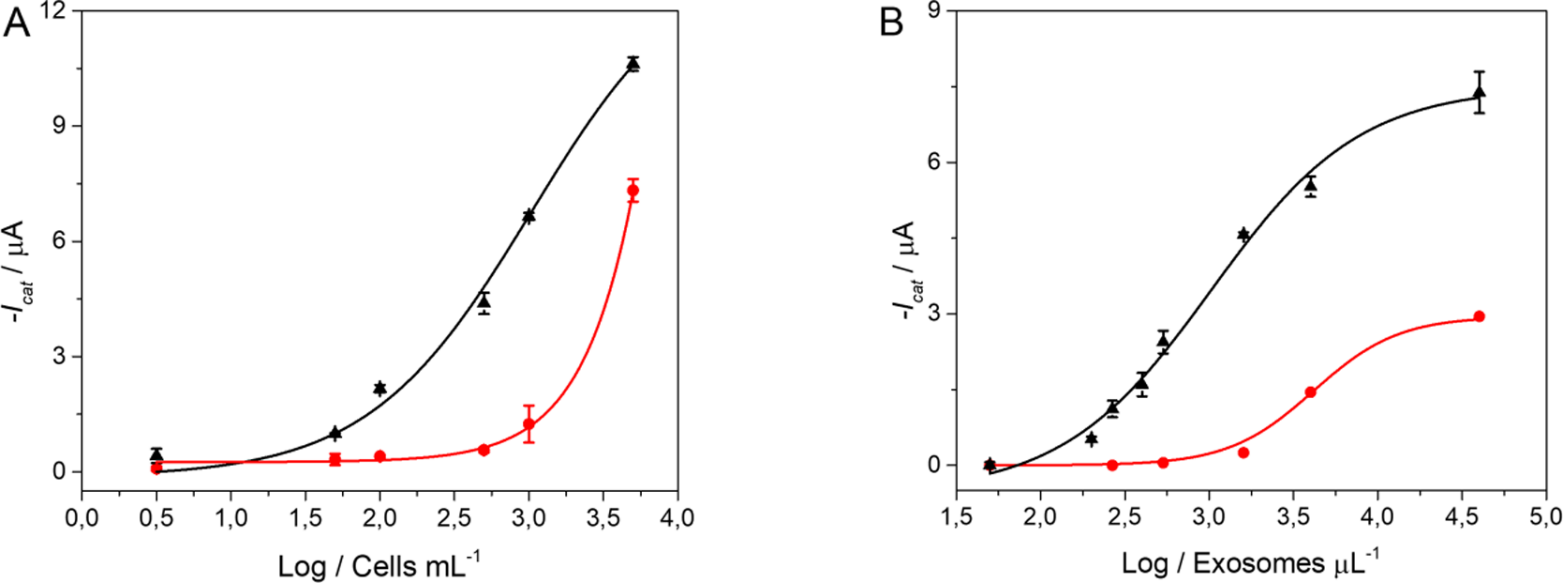
Electrochemical genosensing of GAPDH transcripts from (A) MCF7 cells ranging from 50 to 5000 cells mL^–1^ and (B) their exosomes ranging from 100 to 4.0 × 10^4^ exosomes μL^–1^, according to NTA counting. In all cases, the cells and exosomes were lysed preconcentrated by IMS using antiCD81-MPs (▲) and antiCD326-MPs (●), followed by double-tagging RT-PCR on poly(dT)-MPs. The error bars show the standard deviation for *n* = 3.

The calibration plots for the detection of GAPDH transcripts from MCF7 cells and its derived exosomes are comparatively shown in Figure 3. Two different immunomagnetic separation (IMS) approaches were tested. First, IMS of MCF7 cells by using antiCDX-MPs (where CDX is any of CD81 or CD326 biomarkers), followed by the double-tagging RT-PCR and electrochemical genosensing. Thus, different concentrations of MCF7 cells ranging from 50 to 5000 cells mL^–1^ were evaluated for the calibration plot. The electrochemical responses were fitted using nonlinear regression (four-parameter logistic equation, GraphPad prism software) (Figure 3, panel A). The limit of detection (LOD) of 45 cells mL^–1^ (*r*^2^ = 0.996) and 67 cells mL^–1^ (*r*^2^ = 0.998) was reached for cells immunocaptured by using CD81 and CD326 biomarkers, respectively. Although the strategy was able to clearly detect cells by GAPDH transcript amplification and improved analytical simplification, these LOD values are not suitable for applications in breast cancer diagnosis, since the clinical CTC count assay approved by the U.S Food and Drug Administration (FDA) must be smaller than 5 cells per 7.5 mL^–1^.^7^ At this point, considering the performance of the proposed genosensor, the study was further focused on cancer-related exosomes. These extracellular vesicles are considered as new biomarkers for the detection of cancer in early stages, since it is related to cell-to-cell communication and increased in cancer cells.^11,29^

The IMS of exosomes (ranged from 100 to 4.0 × 10^4^ exosomes μL^–1^) derived from the MCF7 cell line was performed on antiCD81-MPs and/or antiCD326-MPs followed by the double-tagging RT-PCR for the specific GAPDH transcripts on poly(dT)-MPs and subsequent electrochemical genosensing. The electrochemical responses were fitted using nonlinear regression (four-parameter logistic equation, GraphPad prism software) (Figure 3, panel B). The LOD of 415 exosomes μL^–1^ (*r*^2^ = 0.991) and 1225 exosomes μL^–1^ (*r*^2^ = 0.980) were reached using CD81 and CD326 biomarkers, respectively. Although the expression of CD326 is much lower than that of CD81, this effect is minimized by using the positive selection of CD326 exosomes by IMS and preconcentration, followed by double-tagging end-point RT-PCR at 32 cycles for GAPDH, which improves the sensitivity of the approach. IMS of exosomes improves analytical simplification, avoiding ultracentrifugation or other separation steps and have the advantage of a specific capture of exosomes by epithelial breast cancer biomarker, which is currently used in most of the CTC-enrichment methods such as CellSearch.^7^ Since the number of exosomes in plasma ranges from 10^5^ to 10^9^ exosomes μL^–1^,^30^ the LOD for double-tagging RT-PCR based on GAPDH transcripts coupled with electrochemical genosensing was feasible and reliable to detect and quantify cancer-related exosomes.

In this approach, the exosomes are specifically isolated and preconcentrated by IMS, while the double-tagging RT-PCR is used as a strategy to amplify the signal and thus improve the LOD by an order of 2, compared to previous studies (from 1 × 10^5^ to 4 × 10^2^ exosomes μL^–1^).^31,32^ For this reason, in this approach, a common and ubiquitous transcript based on GAPDH was selected for the double-tagging amplification,^33^ while the previous studies were based on a second labeled antibody in a sandwich immunosensing format^27,31^ or the intrinsic alkaline phosphatase enzyme activity in exosomes.^32^ The main shortcoming is that in this approach, the unique source of specificity is provided by the antibody on the MPs during the IMS.

The LOD obtained in this work was better in analytical performance than in fluorescence,^34^ electrochemical,^35^ and surface-enhanced Raman scattering^36^ devices, and comparable to other reported approaches, such as rolling circle amplification^37^ and microfluidic graphene oxide-based^38^ detection.

### Electrochemical Magneto-Genosensing of Transcripts from Exosomes of Breast Cancer Patients

**Figure 4:**
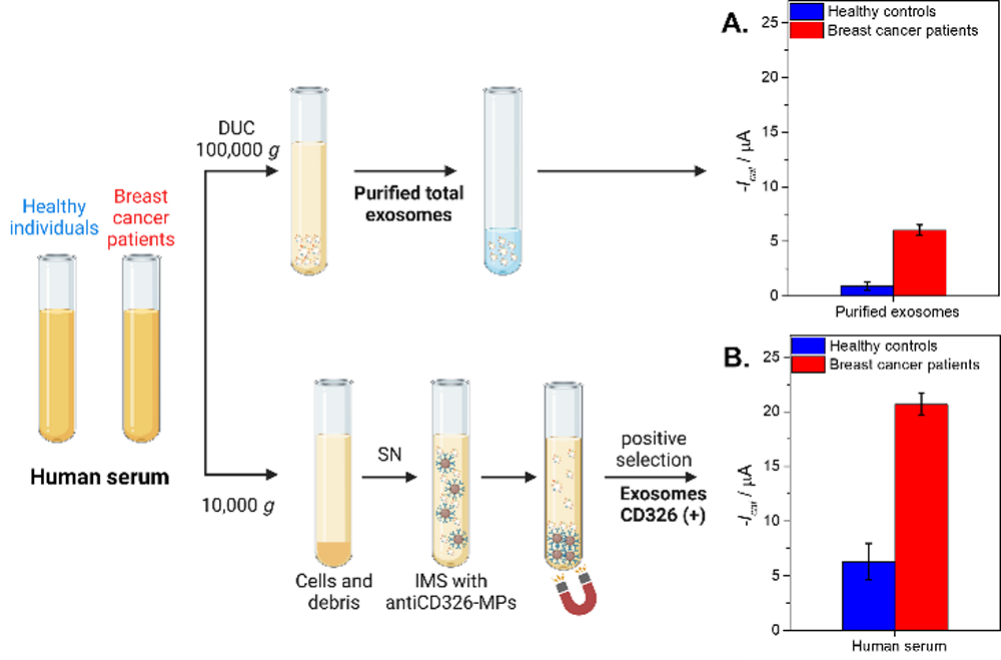
Panel A shows the control of the purified total exosome population obtained by ultracentrifugation (100,000 × *g*) normalized according to the protein content (0.33 μg per assay). Panel B. Electrochemical genosensing of CD326+ exosomes from 1 mL of cell-free undiluted human serum (centrifuged at 10,000 × *g*) based on immunomagnetic separation with antiCD326-MP and further GAPDH transcripts detection. The whole procedure is also shown in Figure 1. In all cases, serum-derived exosomes from healthy controls (*n* = 10, pooled) and breast cancer (*n* = 10, pooled) patients were processed. The error bars show the standard deviation for *n* = 3. The raw amperograms are also shown in Figure S4 (Supplementary Data). Created with BioRender.com.

The performance of the double-tagging RT-PCR on MPs and electrochemical genosensing was evaluated in serum-derived exosomes from healthy and breast cancer patients. The procedure and the results are depicted in Figure 4. All experimental parameters are described in S7 (Supplementary Data). First, the GAPDH expression was evaluated in purified exosomes (without preconcentration on MPs) derived from healthy controls and breast cancer patients (Figure 4, panel A), normalized per micrograms of exosomes (BCA protein assay results are detailed in S7, Supplementary Data). In this approach, the specific IMS and as such the positive selection of CD326 exosomes (Figure 4, panel B) were replaced by a nonspecific physical isolation (ultracentrifugation at 100,000 × *g*). In this case, the approach is based on amplification and detection through a nonspecific GAPDH biomarker. Then, the double-tagging RT-PCR for the specific GAPDH transcripts on poly(dT)-MPs was performed using 0.33 μg per assay of serum-derived exosomes from both groups of samples, followed by subsequent electrochemical genosensing detection. The results of this analysis are shown in Figure 4, panel A. The results suggested that breast cancer patients overexpress GAPDH in total exosomes (6.7-fold) and can be well discriminated from healthy individuals.

Next, in order to achieve the analytical simplification, IMS of exosomes directly from human serum by using antiCD326-MPs was performed, followed by RNA extraction and PCR on poly(dT)-MPs of primer specific for GAPDH transcript labeled with DIG/BIO tags and subsequent electrochemical genosensing. In this case, the approach is based on the specific capture of exosomes by the CD326 epithelial cancer-related biomarker and further detection through a nonspecific GAPDH mRNA biomarker. The electrochemical genosensor is performed in 1 mL of human serum only pretreated by a short centrifugation pulse at 10,000 × *g* (to eliminate any remaining cells or particulate debris), followed by IMS with antiCD326-MP and electrochemical magneto-genosensing, as depicted in Figure 1.

The results are presented in Figure 4, panel B. To confirm the significance of the differences in the value for the healthy control and breast cancer patient samples, a one-tailed p-test (Hi > Ho) at a 95% significance level was performed, Hi being hypothesis and Ho the null hypothesis (as detailed in S7, Supporting Data). Notably, a significant overexpression (3.3-fold) of GAPDH on the immunocaptured CD326 positive exosomes in breast cancer samples when compared with serum-derived exosomes from healthy individuals.

The signal obtained from the CD326-positive exosomes from healthy individuals is probably due to some biomarkers such as CD326 that may also exist on the surface of the nontumorigenic cell-derived exosomes.^39^ Thus, it is expected that exosomes derived from healthy individuals also contain low amounts of CD326 biomarkers in exosomes, but at increased levels in cancer-related exosomes from various carcinomas.^40^ Magnetic particles used for exosome separation avoid the amplification of free mRNA that can be present in the serum samples. As mentioned in previous publications,^41^ the expression of GAPDH may vary in several situations, given that it is a multifunctional protein involved in more than ten functions in mammalian cells. Although GAPDH has been considered as a housekeeping gene in several studies for gene expression normalization, its expression can vary in diseases in which the metabolic state of the cells is altered.^41^ To the best of the authors’ knowledge, this is the first study that reports an overexpression of the GAPDH gene on serum-derived exosomes from breast cancer patients. This is in accordance with the highly expressed GAPDH in breast cancer cells.^42^

## Conclusions

Early diagnosis of breast cancer by standard techniques remains a difficult task due to the low specificity, availability, and high cost, added to the lack of specific symptoms in the early stage and the small size of the primary tumor. The study of novel biomarkers including exosomes are currently under intense investigation. Here, a double-tagging RT-PCR on magnetic beads and electrochemical genosensing demonstrated high sensitivity and specificity for GAPDH gene expression based on specific epithelial CD326 cancer-related exosomes, being able to detect the transcripts produced by as low as 1225 exosomes μL^–1^.

Although further studies should be done with samples in early stages of cancer, our data clearly suggest the GAPDH expression in total exosomes from human serum from healthy and breast cancer patients, which revealed that the GAPDH gene is overexpressed by 6.7-fold in breast cancer women at stage IV, when compared to the healthy controls. Also, CD326 (+) exosomes specifically immunocaptured expressed the GAPDH gene as high as 3.3-fold, when compared to the healthy controls. Interestingly, the positive selection of exosomes expressing CD326 by using immunomagnetic separation provides an effective strategy to separate exosomes with higher efficiency than ultracentrifugation, achieving a mild and specific approach for the isolation and preconcentration a subpopulation of exosomes. This strategy is amenable with point-of-care diagnosis since MPs can be easily integrated in different IVD platforms, including biosensing devices.

In conclusion, although further clinical validation should be performed with a higher number of samples at early stages, the significant increase of the GAPDH transcript content in exosomes from patients compared to healthy individuals envisages its role as a putative biomarker for breast cancer diagnostics and monitoring of metastatic disease. This work thus shows a promising strategy for being implemented at primary health care in low-resource settings based on a minimally invasive liquid biopsy.

## Supporting Information Available

Chemicals and biochemicals; buffers and solutions; cell culturing, exosome isolation, and purification from MCF7 cell lines; immobilization of exosomes and antibodies on magnetic particles; Immobilization of exosomes on magnetic particles; covalent immobilization; characterization of the exosomes derived from the MCF7 breast cancer cell line; electrochemical magneto-genosensing; RNA integrity analysis and DNA sequencing; immunomagnetic separation of the exosomes from undiluted human serum; immunomagnetic separation of the exosomes from undiluted human serum; detection of GAPDH transcripts from purified exosomes; and double-tagging RT-PCR on magnetic beads (PDF)

## Abbreviations

CTC: circulating tumor cell
EpCAM: epithelial cell adhesion molecule
GAPDH: glyceraldehyde-3-phosphate dehydrogenase
IMS: immunomagnetic separation
RT-PCR: reverse transcription polymerase chain reaction.
