# Supplementary material for: Electrochemical Genosensing of Overexpressed GAPDH Transcripts in Breast Cancer Exosomes
Source: Anal Chem. 2023 Jan 23;95(4):2487–95. doi: 10.1021/acs.analchem.2c04773 (PMC9893220; doi:10.1021/acs.analchem.2c04773)
Supplement: Supplementary file 1 — ac2c04773_si_001.pdf [file ac2c04773_si_001.pdf]

Electrochemical genosensing of overexpressed GAPDH transcripts in breast cancer exosomes

Supplementary data

Arnau Pallares-Rusiñol[1, 2, †], Silio Lima Moura[1, †], Mercè Martí[2] and Maria Isabel Pividori[1, 2]

[1]*Grup de Sensors i Biosensors, Departament de Química, Universitat Autònoma de Barcelona, 08193, Bellaterra, Spain*

[2]*Biosensing and Bioanalysis Group, Institute of Biotechnology and Biomedicine, Universitat Autònoma de Barcelona, Bellaterra, 08193, Spain*

[†] *These authors have contributed equally to this work*

Corresponding author:

Prof. María Isabel Pividori
Grup de Sensors & Biosensors
Unitat de Química Analítica
Universitat Autònoma de Barcelona
Edifici Cn. Campus UAB
08193, Bellaterra, Barcelona
Corresponding author: Isabel Pividori
E-mail: isabel.pividori@uab.cat; Fax: +34 93 581 2379; Tel: +34 93 581 2806

Supplementary data

S1. Experimental

Chemicals and biochemicals

Magnetic particles (MPs) tosylactivated (Dynabeads M450 Tosylactivated, ref. 14013), MPs modified with EpCAM (also known as CD326) antibody (Dynabeads Epithelial Enrich, ref. 16102), MPs modified with poly(dt) (polydT-MPs, Dynabeads Oligo(dT), ref. 61002), MPs modified with streptavidin (strep-MPs, Dynabeads MyOne Streptavidin T1, ref. 65601) and mouse monoclonal antibody antiCD81 (ref. 10630D) were purchased from Thermo Fisher Scientific (MC, US). Mouse monoclonal antibody antiEpCAM (ref. ab7504) and Cy®5 fluorophore dye (anti-mouse, ref. ab97037) were purchased from Abcam (Cambridge, GB). Antidigoxigenin-horseradish peroxidase Fab fragments (antiDIG-HRP, Ref. 11207733910) was purchased from Roche Diagnostics (Basel, CH).

Taq DNA polymerase (ref. 18038067), M-MLV reverse transcriptase (ref. 28025013) and RNaseOUT Recombinant ribonuclease Inhibitor (ref. 10777019), Total exosome RNA and protein isolation kit (ref. 4478545), DTT Solution 0.1M (ref. Y00147), FS Buffer 5x (ref. Y02321) and GeneJET Gel Extraction and DNA Cleanup Micro Kit (ref. K0831) were purchased from Thermo Fisher Scientific. Standard reaction buffer 10x with $MgCl_2$ (ref. 20.034-4182) was purchased from Biotools (Madrid, ES). Deoxynucleotide Mix 10mM (ref. D7295) was purchased from Sigma-Aldrich. The primers for the double-tagging PCR were selected for the specific amplification of GAPDH (glyceraldehyde 3-phosphate dehydrogenase) and were purchased from Sigma-Aldrich. The sequence for the digoxigenin-modified forward primer (DIG-Fw) was 5'-[DIG] CTTCTTTTGCGTCGCCAG; while the sequence for the biotin-modified reverse primer (BIO-Rev) was 5'-[BIO] AGCCCCAGCCTTCTCCA. All solutions were prepared with ultrapure MilliQ water (Millipore® System, resistivity 18.2 MΩ·cm) and solutions used in RNA preparation were RNase-free by treatment with 0.1% DEPC.

Buffers and solutions

Diethyl pyrocarbonate (DEPC, ref. D5758), ethylenediaminetetraacetic acid (EDTA, ref. E9884), glycine (ref. 410225), hydroquinone (ref. H9003), hydrogen peroxide (ref. 1072090500), lithium chloride (ref. 746460), lithium dodecyl sulfate (LiDS, ref. L9761), tris(hydroxymethyl)aminomethane (TRIS, ref. 252859), Tween 20 (ref. P9416) were purchased from Sigma-Aldrich (Merck KGaA, DE). DL-Dithiothreitol (DTT, ref. 46819) was purchased from Fluka (Thermo Fisher Scientific). All buffer solutions used

in RNA extraction were prepared with 0.1% diethylpyrocarbonate (DEPC) treated with Ultrapure water (Millipore® System, resistivity 18.2 MΩ cm) to prevent RNA degradation.

The composition of the solutions was:

- Tris 1x buffer: 0.1 mol L^{-1} TRIS-HCl, 0.15 mol L^{-1} NaCl, pH 7.4.
- Tris blocking buffer: 2% w/v BSA, 0.1% w/v Tween 20, 5 mmol L^{-1} EDTA, 0.1 mol L^{-1} TRIS-HCl, 0.15 mol L^{-1} NaCl, pH 7.4.
- ePBS buffer: 0.1 mol L^{-1} Na_2HPO_4, 0.1 mol L^{-1} KCl, pH 7.0.

For the RNA extraction with polydT-MPs, the solutions were:

- RNase-free water: 0.1% v/v DEPC.
- Binding Buffer: 20 mmol L^{-1} TRIS-HCl, pH 7.5, 1.0 mol L^{-1} LiCl, 2 mmol L^{-1} EDTA.
- Lysis/Binding buffer: 100 mmol L^{-1} Tris-HCl, 500 mmol L^{-1} LiCl, 10 mmol L^{-1} EDTA, 1% LiDS, 5 mmol L^{-1} DTT.
- Washing buffer A: 10 mmol L^{-1} TRIS-HCl, pH 7.5, 0.15 mol L^{-1} LiCl, 1 mmol L^{-1} EDTA, 0.1% LiDS.
- Washing buffer B: 10 mmol L^{-1} TRIS-HCl, pH 7.5, 0.15 mol L^{-1}, LiCl, 1 mmol L^{-1} EDTA.

S2. Cell culturing, exosome isolation and purification from MCF7 cell line

MCF7 breast cancer cell line (ATCC, ref. HTB-22) was used. Expansion of cell population was carried out from 1,000,000 cells in T-175 flask containing 32 mL of Dulbecco's Modified Eagle Medium (DMEM, ref. 31966-047, Thermo Fisher Scientific), supplemented with 10% exosome-depleted fetal bovine serum (FBS, ref. 12007C, Sigma-Aldrich), 100 U mL^{-1} penicillin-streptomycin (ref. 15140122, Thermo Fisher Scientific). The temperature was maintained at 37°C in humidified, concentrated CO_2 (5%) atmosphere. Once cells reached approximately 95% confluence on T-175 flask, the culture medium was removed and stored at -21ºC until to exosome isolation.

Exosomes were purified according to *Théry et al.*[1] with minor changes. The supernatant of the cell culture from MCF7 breast cancer cell line, or from human serum was subjected to differential centrifugation as follow: 300 x *g* for 10 minutes (removal of residual cells), 2,000 x *g* for 10 minutes and 10,000 x *g* for 30 minutes (removal of cellular

debris). Then, a Beckman Coulter Optima L-80XP Ultracentrifuge at 100,000 x *g* for 60 minutes with a 70Ti rotor to pellet exosomes. After that, the supernatant was carefully removed, and crude exosome-containing pellets were resuspended in 1 mL of Tris 1x buffer (pH 7.4, 0.22 µm sterile-filtered) and pooled. A second round of same ultracentrifugation setting was carried out, and the resulting exosome pellet resuspended in 500 µL (per 100 mL of supernatant) of Tris 1x buffer (pH 7.4, 0.22 µm sterile-filtered), and storage at -80°C. All centrifugation steps performed at a temperature of 4°C.

The exosomal protein content was determined by using Pierce BCA Protein Assay Kit (ref. 23227, Thermo Fisher Scientific), following the manufacturer protocol, using bovine serum albumin (BSA) standards in Tris 1x buffer. The spectrophotometric measurements were done at 562 nm.

S3. Immobilization of exosomes and antibodies on magnetic particles

Dynabeads M450 tosylactivated superparamagnetic particles (MPs, 4.5 µm in diameter) has a core of iron oxide salt encapsulated by a polystyrene polymer, which has a polyurethane external layer with the p-toluenesulfonate group[2]. It is a good leaving group, which allows an S_N2 reaction to occur in the presence of a nucleophile[3,4]. A nucleophilic reaction by an antibody, protein, peptide, or glycoprotein removes and replaces the sulfonyl ester groups from the polyurethane layer.

Two different approaches were used, as depicted in Figure S1. The first one involves the direct covalent immobilization of exosomes on magnetic particles (Fig. S1, panel A). The second approach is based on the covalent immobilization of the antibodies for a further immunomagnetic separation (IMS) of exosomes (Fig. S1, panel B).

Immobilization of exosomes on magnetic particles

The immobilization of exosomes on Dynabeads M450 tosylactivated superparamagnetic particles (MPs) (Fig. S1, panel A) were performed as follows: S1 x 10^{10} exosomes were added to 40 µL (1.6 x 10^7 MPs) Dynabeads M450 tosylactivated. The reaction kinetics are increased by adding 0.1 mol L^{-1} borate buffer, pH 8.5, in order to ensure the nucleophilic reaction by the amine group. The incubation step was performed overnight with gentle shaking at 4ºC. After that, 0.5 mol L^{-1} glycine solution was added to ensure the blocking of the any remaining tosylactivated groups, by an incubation for 2 h at 25ºC. After that, the exosomes-modified magnetic particles (exosomes-MP) were resuspended in 160 µL of Tris 1x buffer in order to achieve 1 x 10^6 MPs per 10 µL. The exosomes-MP were maintained at 4ºC until use and remain stable on MPs up to two months.

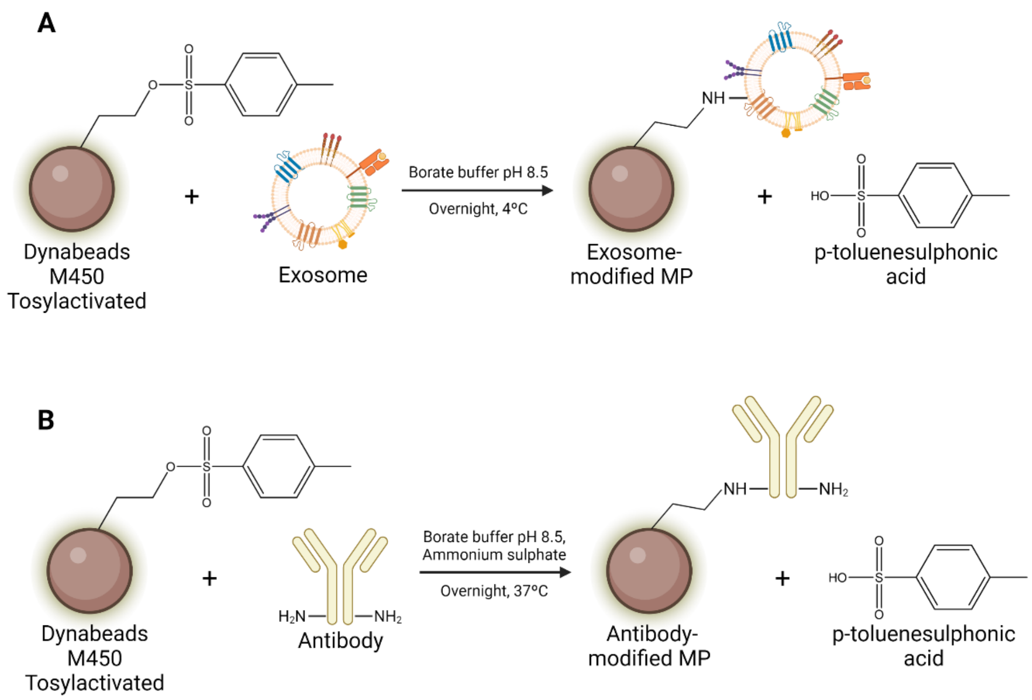

Figure S1. Covalent immobilization of (A) exosome or (B) antibody on Dynabeads® M450 tosylactivated. Created with BioRender.com.

Immobilization of antibodies on magnetic particles

The CD81 antibody (15 µg mL^{-1}, as previously optimized[5]) was added to 55 µL (2.2 x 10^7 MPs) Dynabeads M450 tosylactivated (Fig. S1, panel B). The reaction kinetics are increased by adding 0.1 mol L^{-1} borate buffer pH 8.5 and 3 mol L^{-1} ammonium sulphate in borate buffer. The incubation step was performed overnight (18-20h) with gentle shaking at 37ºC. After that, a blocking step with 0.5 mol L^{-1} glycine solution was performed for 2 h to ensure the blocking of the any remaining tosylactivated groups. After that, the antibody-modified magnetic particles (herein, antiCDX-MPs, where antiCDX = antiCD81) were resuspended in 220 µL (10 µL per assay to give 1 x 10^6 particles per assay) of Tris 1x buffer.

It is important to highlight that in this procedure it was no possible to achieve the immobilization of antiCD326 antibody on MPs. Therefore, commercially modified particles with EpCAM were used. EpCAM corresponds to CD326 (Cluster of Differentiation nomenclature), so in this work, antiCD326-MPs are equally referred to commercial antiEpCAM-MPs.

S4. Characterization of the exosomes derived from MCF7 breast cancer cell line

Characterization of exosomes by nanoparticle tracking analysis and cryogenic transmission electron microscopy

The size distribution and concentration of exosomes were measured by nanoparticle tracking analysis (NTA) using the NanoSight LM10-HS system with a tuned 405 nm laser (NanoSight Ltd., GB). The purified exosomes were diluted in sterile-filtered TRIS buffer (50- to 100-fold). Nanosight NTA Software analyzed raw data videos by triplicate during 60 s with 50 frames s^{-1} and the temperature of the laser unit set at 24.8ºC. For the cryogenic transmission electron microscopy (Cryo-TEM), the exosomes (2.0 x 10^9) were directly laid on Formvar-Carbon EM grids and frozen in ethanol. TEM images were collected by a Jeol JEM 2011 (JEOL USA Inc., US) transmission electron microscope at an accelerating voltage of 200 kV. Exosomes were maintained at -182°C during the whole process.

Characterization of exosomes by bead-based flow cytometry and confocal microscopy

The analysis of the molecular biomarkers expressed in MCF7 cell line was firstly carried out by flow cytometry. The presence of the CD81 and CD326 biomarkers was investigated. The indirect labeling of 2 x 10^5 cells was performed by incubation of 100 µL (5 µg mL^{-1}) of the antibodies antiCD81 and antiCD326, for 30 min with gentle shaking at 25ºC. After that, three washing steps with Tris 1x buffer containing 0.5% BSA were performed. Afterwards, 100 µL (2 µg mL^{-1}) of antimouse-Cy5 antibody (far-red fluorescent dye, excitation 647 nm, emission 665 nm) was incubated for 30 min in the darkness with gentle shaking at 25 ºC. The labeled cells were resuspended in 200 µL of Tris 1x containing 0.5% BSA. The same procedure of labeling was performed in the case of the exosomes derived from MCF7 breast cancer cell line, but in this approach, and due to their size and resolution of the technique, the exosomes were firstly immobilized on the surface of MPs, as described on To achieve that, 3.5 x 10^{10} exosomes were covalently immobilized on 1.6 x 10^7 MPs, as described in S3, followed by the indirect labeling as described above, with antiCD81 or antiCD326.

The same batch of cells and exosomes analyzed by flow cytometry were subjected for confocal microscopy imaging for the study of the binding pattern of antibodies. In the case of cells, the cellular DNA was stained previously (before labeling

with antibodies) with Hoechst dye (blue fluorescent dye, excitation 350 nm, emission 490 nm).

S5. Immunomagnetic separation, double-tagging reverse transcription PCR of GAPDH transcripts and electrochemical genosensing

Immunomagnetic separation of the cells and exosomes

The IMS of the cells exosomes was performed by antiCDX-MPs (being CDX any of CD81 or CD326 biomarkers) (containing 1 × 10^6 MPs per tube), and 100 µL of MCF-7 cells (concentration ranging from 50 to 5.000 cells mL^{-1}) or exosomes (concentration ranging from 100 to 4.0 x 10^4 exosomes μL^{-1}), which were simultaneously incubated for 30 min with gentle shaking at 25°C, followed by three washing steps with Tris 1x buffer containing 0.5% BSA. The coated antiCDX-MPs were resuspended in 1.0 mL of Lysis/Binding buffer. Then, they were disrupted by pipetting up and down a couple of times to ensure a complete lysis. In order to ensure sample homogenization, the lysate was passed through a 21-gauge needle using a 2.0 mL syringe. Then, the lysate and antiCDX-MPs were separated by using a magnet plate separator, an antiCDX-MPs pellet on the bottom tube is formed, and the lysate is transferred to another tube.

Double-tagging RT-PCR on magnetic beads

The lysate of the cells or exosomes was incubated with 15 µL of poly(dT)-MPs (7 x 10^7 MPs) for 15 min under gentle shaking at 25ºC. Finally, the mRNA-coated MPs were washed with 500 µL of washing buffer A, followed by washing with 500 µL of washing buffer B, and finally resuspended with 100 µL of DEPC-treated water. The suspension of RNA-poly(dT)-MPs was stored on ice and immediately used.

The RNA reverse transcription (RT) was carried out on poly(dT)-MPs with Moloney Murine Leukemia Virus (M-MLV) reverse transcriptase. The RNA-poly(dT)-MPs was placed in a magnet tube separator, allowing to discard the supernatant (DEPC-treated water) from the RNA-poly(dT)-MPs that remain pelleted at the bottom of the tube for subsequent RT. The poly(dT)-MPs were incubated with 10 nmol of dNTPs mix for 5 min at 65 ºC and cooled on ice for 1 min. After that, a mix containing 200 nmol of DTT, 40 U of RNaseOUT inhibitor and 1x First Strand Buffer was added and incubated at 37 ºC for 2 min. Finally, 200 U of M-MLV reverse transcriptase were added and incubated for 50 min at 37 ºC, and 15 min at 70 ºC for inactivating the reaction. The cDNA was stored at -21ºC until use.

The double-tagging polymerase chain reaction (PCR) was performed in 15 µL of reaction mixture containing the cDNA as sample in order to obtain the GAPDH amplicons

doubly labelled with biotin and digoxigenin. Each reaction mixture contained 7.5 pmol of each primer (DIG-Fw and BIO-Rev), 3.75 nmol of each deoxynucleotide triphosphate (dNTPs) and 3U of Taq polymerase. The reaction was carried out in a buffer with 7.5 mmol L^{-1} Tris buffer (pH 9.0), 5.0 mmol L^{-1} KCl, 2.0 mmol L^{-1} $(NH_4)_2SO_4$ and 0.2 mmol L^{-1} $MgCl_2$ as a cofactor of the enzyme. The reaction mixture was exposed to an initial step at 95 °C for 3 min followed by 32 cycles of 95 °C for 30 s, 61 °C for 30 s, 72 °C for 30 s, and a last step of 7 min at 72 °C.

Multiple negative samples for the RT and PCR, which contained all reagents except mRNA or cDNA were tested. The performance of the double-tagging PCR was analyzed with agarose gel electrophoresis followed by DNA sequencing analysis. The agarose gel electrophoresis was done with 2% agarose gel in TAE buffer containing 1×GelRed dye and a molecular weight marker of DNA fragments ranged from 100 to 1000 base pair (bp), that was used as size amplicon control. The DNA bands were visualized by UV transillumination, expecting a single DNA band at 371 bp in all samples. DNA bands obtained with samples of MCF-7 cells and exosomes were purified with GeneJET kit and analyzed, as described in S6 (Supp. Data).

Optimization of RT-PCR amplification cycles

As aforementioned, the detection of exosomes is a challenging task due to the low concentration in biological samples. Moreover, an intrinsic characteristic of the exosomes is the low RNA content compared to cells[6]. In order to increase the sensitivity of the approach, the double-tagging RT-PCR was optimized towards the number of cycles required for GAPDH transcript detection in exosomes. The cellular GAPDH transcript detection was used for comparison purposes. The double-tagging RT-PCR was performed with 28, 32, 36 and 40 cycles.

The double-tagged amplicons were submitted in parallel to gel electrophoresis and measured by electrochemical magneto genosensing. Negative controls were performed with all reagents, omitting the RNA (from cells and/or exosomes). Figure S2, panel A shows the gel electrophoresis for cellular and exosomal GAPDH amplicons. While the GAPDH amplicons from MCF7 cells were observed in all PCR cycles tested, the amplicons for exosomes are only evidenced after 36 cycles. Then, the GAPDH amplicons from exosomes were subjected to electrochemical genosensing. As expected, the electrochemical genosensing revealed that in the exosomes the GAPDH transcript was also amplified in all PCR cycles tested (Fig. S2, panel B). However, the signal-to-noise ratio for the detection of the GAPDH amplicons is affected substantially by the increase in the PCR cycles (Fig. S2, panel B inset). Probably, this is due to a larger

number of dimers formed as the PCR cycles increases, and the best signal-to-noise ratio was obtained with 32 cycles of PCR, as shown in the inset of the Figure S2, panel B, since higher cycles shows saturation of the magnetic particles with the product. This result also highlights the higher sensitivity of the double-tagging RT-PCR coupled to the electrochemical detection compared with the gel electrophoresis.

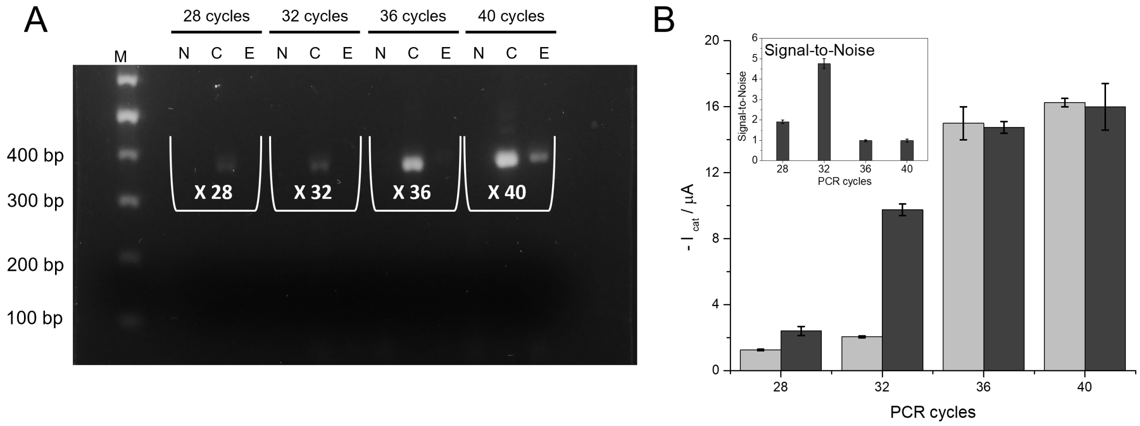

Figure S2. Evaluation of the number of cycles in the double-tagging RT-PCR for GAPDH transcript detection from MCF7 cells and their exosomes, detecting by gel electrophoresis (panel A) and electrochemical genosensing (panel B). Panel A shows the gel electrophoresis with identified lanes for negative (N), cells (C) and exosomes (E) in the respective PCR cycles. Lane M corresponds to 100 bp DNA size marker. The corresponding signals for the double-tagging RT-PCR electrochemical genosensing are shown in panel B (black bars), also showing the non-specific adsorption (grey bars). The inset shows the signal-to-noise current. The error bars show the standard deviation for n = 3.

Electrochemical magneto-genosensing

The procedure for the detection of the BIO-DIG double-tagged PCR product is based on the immobilization on streptavidin-modified magnetic particles and its electrochemical detection with specific antibody for digoxigenin modified with HRP. The magneto-actuated electrochemical genosensing (Fig. 1, panel C) was performed in tubes and involved the following steps. (i) Immobilization of the amplicons on Strep-MPs. For that, 30 µL of the amplicons diluted 25-fold in Tris 1x buffer were incubated with 7 x 10^7 Strep-MPs during 5 min at 25 ºC. (ii) Labeling with electrochemical reporter. Incubation with 10 µL (130 mU) of antiDIG-HRP in Tris blocking buffer, during 30 min at 25 ºC, followed by three washing steps.

For the electrochemical readout, the strep-MPs coated with the amplicons were separated by using a magnet tube separator, a MPs pellet on the bottom tube is formed, followed by remove of the supernatant. Following, MPs pellet is resuspended in ePBS buffer and a magneto-actuated graphite-epoxy composite (m-GEC) electrode is inserted into tube for remove the MPs pellet onto m-GEC surface, which is transferred into an

electrochemical cell and measured by means of amperometry at -100 mV *vs.* Ag/AgCl/$KCl_{(sat.)}$ by using hydroquinone mediator. For that, a standard one compartment three-electrode electrochemical cell is filling with 19.8 mL of ePBS, 100 µL of 400 mmol L^{-1} hydroquinone (HQ) as electrochemical mediator, and 100 µL of 400 mmol L^{-1} H_2O_2 as substrate. A reproducible steady-current was obtained after 60 s. The cathodic current generated by monitoring benzoquinone species directly related with the amount of captured exosomes. The m-GEC surface cleaning procedure was carried out for every experiment. First, the electrode surface was cleaned with absorbent paper, then by an electrochemical treatment by applying a potential of +3 V for 5 s in 0.5 mol L^{-1} H_2SO_4 supporting electrolyte.

S6. RNA integrity analysis and DNA sequencing

A comparative integrity study of RNA from MCF7 breast cancer and purified exosomes was performed. To achieve that, the RNA obtained by lysis of cells and exosomes were processed by classical RNA extraction and purification procedure followed by integrity analysis. The Total Exosome RNA and protein isolation kit were used to obtain RNA from MCF-7 cells (1 x 10^6 cells) and purified exosomes (1 x 10^{10} exosomes), following the manufacturer protocol. The samples were analyzed with Agilent RNA 6000 Nano Kit (ref. 5067-1511) from Agilent Technologies (CA, US) by Genomics Bioinformatics Service (Institute of Biotechnology and Biomedicine, UAB, ES) to characterize and quantify the RNA content.

Figure S3 shows the results of the RNA integrity analysis. Firstly, the quality of the extracted RNA was assessed by the Bioanalyzer RNA integrity numbers (RIN; 1 = totally degraded, 10 = intact). The cellular 18S and 28S ribosomal RNA (rRNA) are the most dominant peaks, and the RIN value was estimated to be 8.0 consistent with a good RNA quality (Fig. S3, panel A). In addition to the rRNA, one broadband (~100 to ~450 nucleotides) for cellular messenger RNA (mRNA) also is displayed. Unfortunately, rRNA nor mRNA peaks were not observed for RNA extracted from exosomes (Fig. S3, panel B). Since the algorithm is based on the ribosomal RNA, previous studies demonstrated that exosomes contain little or no rRNA[7,8] and mRNA[9], RIN values are only valid for cellular RNA quality assessments. It is important to highlight that RNA yield can differ substantially between different RNA isolation methods, which may be related to the low sensitivity of the extraction method.[10]

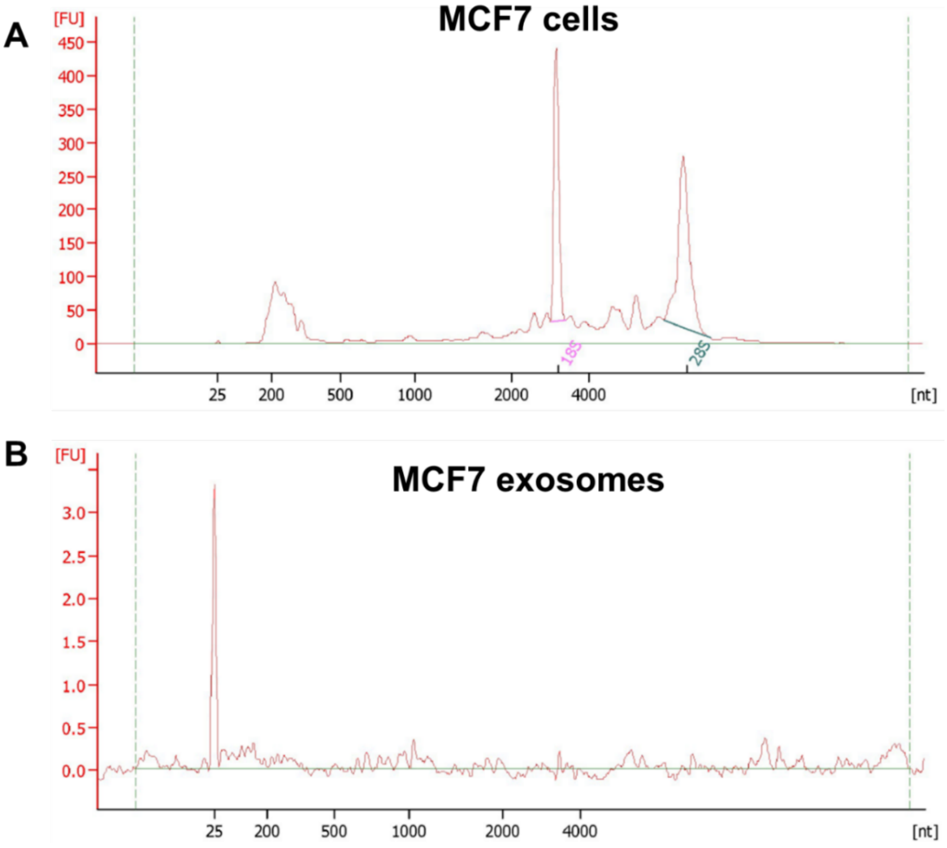

Figure S3. RNA integrity analysis of MCF-7 cells and exosomes samples. Panel A, the sample from MCF-7 cells shows rRNA 18s and 28s bands apear in a ratio 1.2, with a high RNA integrity number (RIN = 8.0). Panel B, the sample from exosomes does not show any rRNA band, the RNA concentration was below the bioanalyzer sensitivity.

Regarding the DNA sequencing analysis, an ABI Prism 3130XL Genetic Analyzer was used, with BigDye Terminator v3.1 Cycle Sequencing Kit (ref. 4336919, Thermo Fisher Scientific), provided by Genomics Bioinformatics Service (Institute of Biotechnology and Biomedicine, UAB, ES). The results were analyzed using Chromas v 2.6.6 (Technelysium Pty Ltd, Brisbane, QLD, AU) and Clustal Omega[11] software, to check the chromatograms and the alignment of both sequences. Then, the amplified sequence was identified using BLAST software[12]. As expected, the DNA sequencing revealed the entire specific GAPDH sequence in both cells and exosomes, with no other enriched fragments. This result demonstrated that both RNA extraction and reverse transcription can be performed on poly(dT)-MPs. Nonetheless, the use of poly(dT)-MPs and the magnetic actuation simplifies the analytical procedure, when compared to the classical procedure for RNA extraction, avoiding the use of separation columns and further centrifugation steps.

This procedure was able to identify the following sequence, that contains the forward primer sequence highlighted in blue color and the reverse primer sequence in red color.

Homo sapiens glyceraldehyde-3-phosphate dehydrogenase (GAPDH), transcript variant 1, mRNA. NCBI Reference Sequence: NM_002046.7.

GCTCTCTGCTCCTCCTGTTCGACAGTCAGCCGCAT**CTTCTTTTGCGTCGCC**
AGCCGAGCCACATCGCTCAGACACCATGGGGAAGGTGAAGGTCGGAGTCAACGG
ATTTGGTCGTATTGGGCGCCTGGTCACCAGGGCTGCTTTTAACTCTGGTAAAGTG
GATATTGTTGCCATCAATGACCCCTTCATTGACCTCAACTACATGGTTTACATGTTC
CAATATGATTCCACCCATGGCAAATTCCATGGCACCGTCAAGGCTGAGAACGGGA
AGCTTGTCATCAATGGAAATCCCATCACCATCTTCCAGGAGCGAGATCCCTCCAA
AATCAAGTGGGGCGATGCTGGCGCTGAGTACGTCGTGGAGTCCACTGGCGTCTT
CACCACCA**TGGAGAAGGCTGGGGCT**CATTTGCAGGGGGGAGCCAAAAGGGTCAT
CATCTCTGCCCCCTCTGCTGATGCCCCCATGTTCGTCATGGGTGTGAACCATGAG
AAGTATGACAACAGCCTCAAGATCATCAGCAATGCCTCCTGCACCACCAACTGCT
TAGCACCCCTGGCCAAGGTCATCCATGACAACTTTGGTATCGTGGAAGGACTCAT
GACCACAGTCCATGCCATCACTGCCACCCAGAAGACTGTGGATGGCCCCTCCGG
GAAACTGTGGCGTGATGGCCGCGGGGCTCTCCAGAACATCATCCCTGCCTCTAC
TGGCGCTGCCAAGGCTGTGGGCAAGGTCATCCCTGAGCTGAACGGGAAGCTCAC
TGGCATGGCCTTCCGTGTCCCCACTGCCAACGTGTCAGTGGTGGACCTGACCTG
CCGTCTAGAAAAACCTGCCAAATATGATGACATCAAGAAGGTGGTGAAGCAGGCG
TCGGAGGGCCCCCTCAAGGGCATCCTGGGCTACACTGAGCACCAGGTGGTCTCC
TCTGACTTCAACAGCGACACCCACTCCTCCACCTTTGACGCTGGGGCTGGCATTG
CCCTCAACGACCACTTTGTCAAGCTCATTTCCTGGTATGACAACGAATTTGGCTAC
AGCAACAGGGTGGTGGACCTCATGGCCCACATGGCCTCCAAGGAGTAAGACCCC
TGGACCACCAGCCCCAGCAAGAGCACAAGAGGAAGAGAGAGACCCTCACTGCTG
GGGAGTCCCTGCCACACTCAGTCCCCCACCACACTGAATCTCCCCTCCTCACAGT
TGCCATGTAGACCCCTTGAAGAGGGGAGGGGCCTAGGGAGCCGCACCTTGTCAT
GTACCATCAATAAAGTACCCTGTGCTCAACCA

S7. Electrochemical magneto-genosensing of transcripts from exosomes of breast cancer patients

Human serum isolation

The human serum samples (healthy and breast cancer patients) were separated from the blood cells using a sterile empty tube without any anticoagulant, leave the tube

in a standing position for about 20-30 minutes for blood to be clotted. After that, centrifugation at 1500 x *g* (20 ºC) for 10 minutes was carried out for removal of residual cells and cellular debris. Following, the human serum (supernatant on top) was carefully removed, freeze at -80 ºC to preserve for further assays.

Detection of GAPDH transcripts from purified exosomes without preconcentration on MPs

This approach (Fig. 4, panel A) is based on amplification and detection through non-specific GAPDH biomarker. Firstly, exosomes were isolated from 1.0 mL of human serum from healthy (n = 10, pooled) and breast cancer (n = 10, pooled) patients by ultracentrifugation and resuspended in Tris 1x buffer, as described in S2 (Supp. Data). Then, the exosomes samples from healthy and breast cancer patients were analyzed with the BCA protein assay kit, and their protein concentrations were estimated to be 235 µg mL^{-1} and 335 µg mL^{-1}, respectively. To normalize the results according to the protein content, 0.33 µg of exosomes from healthy and breast cancer patients were subjected to RNA extraction based on poly(dT)-MPs, followed by double-tagging PCR, and subsequent electrochemical genosensing, as described above.

Immunomagnetic separation of the exosomes from undiluted human serum

Our detection approach was to isolate and detect exosomes from undiluted human serum (healthy and breast cancer patients) directly by immunomagnetic separation (IMS) based on antiCD326-MPs (Fig. 4, panel B). In this case, samples of undiluted human serum from healthy (n=10, pooled) and breast cancer patients (n=10, pooled) were centrifuged at 10,000 x *g* to eliminate possible cell debris remaining in the serum. The IMS of the exosomes was performed with antiCD326-MPs (containing 1 × 10^6 MPs per tube), and 1.0 mL of undiluted human serum, incubated for 30 min with gentle shaking at 25 °C, followed by three washing steps with Tris 1x buffer containing 0.5% BSA. Then, the exosomes-coated antiC326-MPs were resuspended with 100 µL of Tris 1x buffer, stored on ice and immediately used for RNA extraction. The exosomes-coated antiCD326-MPs were resuspended in 1.0 mL of Lysis/Binding buffer. The exosomes were disrupted by pipetting up and down a couple of times to ensure a complete lysis. In order to ensure sample homogenization, the lysate was passed through a 21-gauge needle using a 2.0 mL syringe. Then, the lysate and antiCD326-MPs were separated by using a magnet plate separator, an antiCD326-MPs pellet on the bottom tube is formed, followed by lysate separation, and transferred by another tube.

Double-tagging RT-PCR on magnetic beads

After that, the lysate was incubated with 15 µL of poly(dT)-MPs (75 µg MPs) for 15 min under gentle shaking at 25 ºC. Finally, the mRNA-coated MPs were washed with 500 µL of washing buffer A, followed by washing with 500 µL of washing buffer B, and finally resuspended with 100 µL of DEPC-treated water. The suspension of RNA-poly(dT)-MPs was stored on ice and immediately used for reverse transcription reaction, as for the case of the exosomes derived from MCF7 cells.

The double-tagging polymerase chain reaction (PCR) was also performed as above for the case of the exosomes derived from cell culturing.

Electrochemical magneto-genosensing

The procedure for the detection of the BIO-DIG double-tagged PCR product was also performed as above for the case of the exosomes derived from cell culturing.

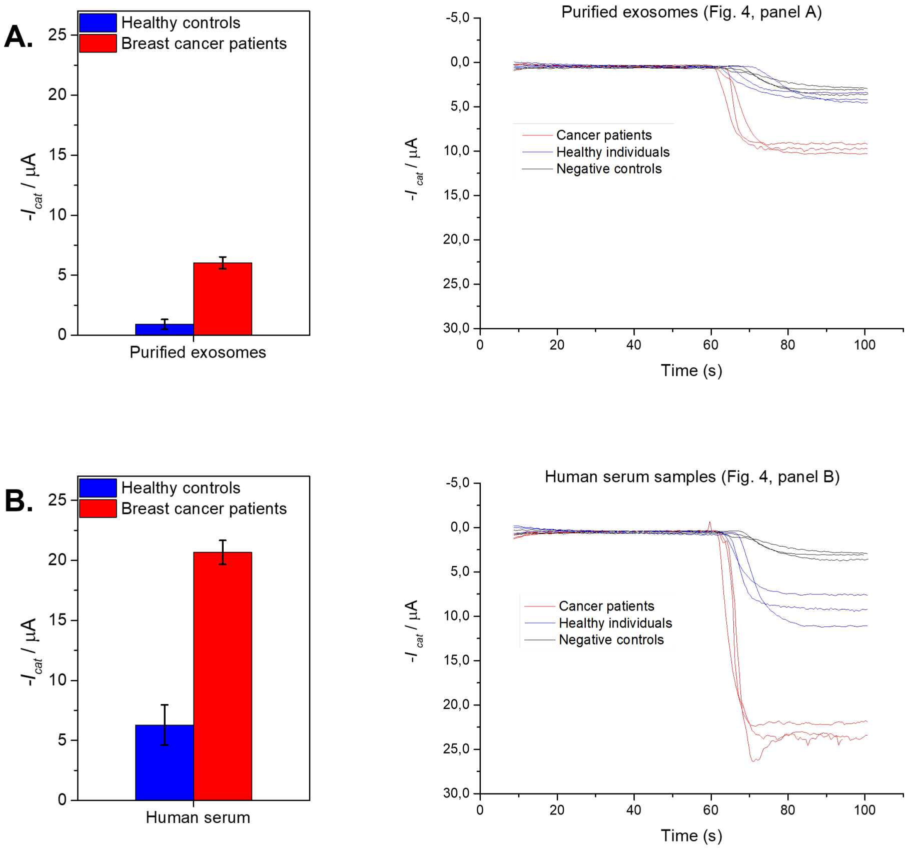

Figure S4. Panel A shows the control of the purified total exosome population obtained by ultracentrifugation (100,000 x g) normalized according to protein content (0.33 µg per assay). Panel B. Electrochemical genosensing of CD326+ exosomes from 1 mL of cell-free undiluted human serum (centrifuged at 10,000 g*) based on immunomagnetic separation with antiCD326-MP and further GAPDH transcripts detection. In all cases, serum-derived exosomes from healthy controls (n = 10, pooled) and breast cancer (n = 10, pooled) patients were processed. The error bars show the standard deviation for n = 3. The raw data for the amperograms are also shown.*

To confirm the significance of the differences in the value for the healthy control and breast cancer patient samples, a one-tailed p-test (Hi > Ho) at a 95 % significance level was performed, being Hi hypothesis and Ho the null hypothesis. The mean value and standard deviations of the electrochemical measurements depicted in Figure S4 were used to calculate the p-values of both comparisons, purified exosomes (Fig. S4, panel A) and human serum samples (Fig. S4, panel B). The calculation was done with data analysis tool, considering equal variances in the samples, obtaining the following results:

Purified exosomes (Fig. S4, panel A): $p = 0.00017 \rightarrow p<0.05$

Human serum samples (Fig. S4, panel B): $p = 0.00011 \rightarrow p<0.05$

S8. References

(1) Théry, C.; Amigorena, S.; Raposo, G.; Clayton, A. Isolation and Characterization of Exosomes from Cell Culture Supernatants and Biological Fluids. *Curr. Protoc. Cell Biol.* **2006**, *30* (1), 3.22.1-3.22.29. https://doi.org/10.1002/0471143030.cb0322s30.

(2) Xu, J.; Mahajan, K.; Xue, W.; Winter, J. O.; Zborowski, M.; Chalmers, J. J. Simultaneous, Single Particle, Magnetization and Size Measurements of Micron Sized, Magnetic Particles. *J. Magn. Magn. Mater.* **2012**, *324* (24), 4189–4199. https://doi.org/10.1016/j.jmmm.2012.07.039.

(3) Hoogenboom, R.; Fijten, M. W. M.; Kickelbick, G.; Schubert, U. S. Synthesis and Crystal Structures of Multifunctional Tosylates as Basis for Star-Shaped Poly(2-Ethyl-2-Oxazoline)S. *Beilstein J. Org. Chem.* **2010**, *6*, 773–783. https://doi.org/10.3762/bjoc.6.96.

(4) Cahiez, G.; Lefèvre, N.; Poizat, M.; Moyeux, A. A User-Friendly Procedure for the Preparation of Secondary Alkyl Chlorides. *Synthesis (Stuttg).* **2012**, *45* (02), 231–236. https://doi.org/10.1055/s-0032-1317927.

(5) Moura, S. L.; Martín, C. G.; Martí, M.; Pividori, M. I. Multiplex Detection and Characterization of Breast Cancer Exosomes by Magneto-Actuated Immunoassay. *Talanta* **2020**, *211*, 120657. https://doi.org/https://doi.org/10.1016/j.talanta.2019.120657.

(6) Chevillet, J. R.; Kang, Q.; Ruf, I. K.; Briggs, H. A.; Vojtech, L. N.; Hughes, S. M.; Cheng, H. H.; Arroyo, J. D.; Meredith, E. K.; Gallichotte, E. N.; Pogosova-Agadjanyan, E. L.; Morrissey, C.; Stirewalt, D. L.; Hladik, F.; Yu, E. Y.; Higano, C. S.; Tewari, M. Quantitative and Stoichiometric Analysis of the MicroRNA Content of Exosomes. *Proc. Natl. Acad. Sci. U. S. A.* **2014**, *111* (41), 14888–14893. https://doi.org/10.1073/pnas.1408301111.

(7) Eldh, M.; Ekström, K.; Valadi, H.; Sjöstrand, M.; Olsson, B.; Jernås, M.; Lötvall, J. Exosomes Communicate Protective Messages during Oxidative Stress; Possible Role of Exosomal Shuttle RNA. *PLoS One* **2010**, *5* (12), e15353. https://doi.org/10.1371/journal.pone.0015353.

(8) Lässer, C.; Seyed Alikhani, V.; Ekström, K.; Eldh, M.; Torregrosa Paredes, P.; Bossios, A.; Sjöstrand, M.; Gabrielsson, S.; Lötvall, J.; Valadi, H. Human Saliva,

Plasma and Breast Milk Exosomes Contain RNA: Uptake by Macrophages. *J. Transl. Med.* **2011**, *9* (1), 9. https://doi.org/10.1186/1479-5876-9-9.

(9) Yuan, T.; Huang, X.; Woodcock, M.; Du, M.; Dittmar, R.; Wang, Y.; Tsai, S.; Kohli, M.; Boardman, L.; Patel, T.; Wang, L. Plasma Extracellular RNA Profiles in Healthy and Cancer Patients. *Sci. Rep.* **2016**, *6* (1), 19413. https://doi.org/10.1038/srep19413.

(10) Eldh, M.; Lötvall, J.; Malmhäll, C.; Ekström, K. Importance of RNA Isolation Methods for Analysis of Exosomal RNA: Evaluation of Different Methods. *Mol. Immunol.* **2012**, *50* (4), 278–286. https://doi.org/10.1016/j.molimm.2012.02.001.

(11) Chojnacki, S.; Cowley, A.; Lee, J.; Foix, A.; Lopez, R. Programmatic Access to Bioinformatics Tools from EMBL-EBI Update: 2017. *Nucleic Acids Res.* **2017**, *45* (W1), W550–W553. https://doi.org/10.1093/nar/gkx273.

(12) Agarwala, R.; Barrett, T.; Beck, J.; et al. Database Resources of the National Center for Biotechnology Information. *Nucleic Acids Res.* **2018**, *46* (D1), D8–D13. https://doi.org/10.1093/nar/gkx1095.
